# Proteomic Responses of Dark-Adapted *Euglena gracilis* and Bleached Mutant Against Light Stimuli

**DOI:** 10.3389/fbioe.2022.843414

**Published:** 2022-03-03

**Authors:** Zhenfan Chen, Zixi Chen, Jiayi Zhu, Jiayi He, Qiong Liu, Hui Zhu, Anping Lei, Jiangxin Wang

**Affiliations:** ^1^ Shenzhen Key Laboratory of Marine Bioresource and Eco-Environmental Science, Shenzhen Engineering Laboratory for Marine Algal Biotechnology, Guangdong Provincial Key Laboratory for Plant Epigenetics, College of Life Sciences and Oceanography, Shenzhen University, Shenzhen, China; ^2^ Key Laboratory of Optoelectronic Devices and Systems of Ministry of Education and Guangdong Province, College of Optoelectronic Engineering, Shenzhen University, Shenzhen, China; ^3^ College of Food Engineering and Biotechnology, Hanshan Normal University, Chaozhou, China; ^4^ Shenzhen-Hong Kong Institute of Brain Science, Shenzhen, China

**Keywords:** *Euglena gracilis*, proteome, chloroplast development, bleached strain, light exposure restoration

## Abstract

*Euglena gracilis (E. gracilis)* has secondary endosymbiotic chloroplasts derived from ancient green algae. Its chloroplasts are easily lost under numerous conditions to become permanently bleached mutants. Green cells adapted in the dark contain undeveloped proplastids and they will develop into mature chloroplasts after 3 days of light exposure. Thus, *E. gracilis* is an ideal model species for a chloroplast development study. Previous studies about chloroplast development in *E. gracilis* focused on morphology and physiology, whereas few studies have addressed the regulatory processes induced by light in the proteome. In this study, the whole-genome proteome of dark-adapted *E. gracilis* (WT) and permanently ofloxacin-bleached mutant (B2) was compared under the light exposure after 0, 12, and 72 h. The results showed that the photosynthesis-related proteins were up-regulated over time in both WT and B2. The B2 strain, with losing functional chloroplasts, seemed to possess a complete photosynthetic function system. Both WT and B2 exhibited significant light responses with similar alternation patterns, suggesting the sensitive responses to light in proteomic levels. The main metabolic activities for the utilization of carbon and energy in WT were up-regulated, while the proteins with calcium ion binding, cell cycle, and non-photosynthetic carbon fixation were down-regulated in B2. This study confirmed light-induced chloroplast development in WT from dark, and also for the first time investigates the light responses of a bleached mutant B2, providing more information about the unknown functions of residual plastids in *Euglena* bleached mutants.

## Introduction

*Euglena gracilis (E. gracilis)* a unicellular green eukaryotic microalga, belonging to the group of euglenids (Excavata), is widely distributed in aquatic environments. *E. gracilis* cells are rich in nutrients with many high-value products, such as paramylon, methionine, vitamins, and fatty acids and has become a new kind of popular product in the market (Mokrosnop, 2018; Gissibl et al., 2019; Kottuparambil et al., 2019). Euglenids exhibit diverse modes of nutrition, including autotrophy, heterotrophy, and mixotrophy with both animal and plant characteristics (Torihara and Kishimoto, 2015).

It is worth mentioning that the chloroplasts of *E. gracilis* are embedded by three membranes as the result of secondary endosymbiosis. The ancestor of *E. gracilis* is known as a heterotrophic animal species and captures green algae resulting in the acquisition of photosynthetic ability by secondary endosymbiosis. *E. gracilis* nuclear genome contains not only genes of green algal origin but also chloroplasts that are identical to chloroplasts found in some green algae (Maruyama et al., 2011; Markunas and Triemer, 2016). However, the chloroplasts in *E. gracilis* seem to be gratuitous for survival. For example, many treatments such as mutagens, heat, and pressure could bleach *E. gracilis*, and permanently lose most, if not all, of their chloroplast genomes but with few impacts on their heterotrophic growth (Heizmann et al., 1976; Osafune and Schiff, 1983). Moreover, the bleached mutants can be induced by the aminoglycosides treatment, such as ofloxacin or streptomycin (Shibata et al., 1954; Tamaki et al., 2020; Tomečková et al., 2020). In our previous study, five bleached mutants of *E. gracilis* treated with ofloxacin were obtained and 12 residual plastid genes of the total chloroplast genome were investigated in both WT and bleached mutants. One of the bleached mutants named B2 lost the ability to accumulate chlorophyll, which was due to many genes of chloroplasts being undetected at the genomic and transcriptional levels, such as *psbE* and *psbK* (Qin et al., 2020).

In the dark, *E. gracilis* plastids become poorly developed without green pigments and can be recovered into photosynthetically capable chloroplasts after light exposure restoration for 3 days (Stern et al., 1964). This process has been well studied since the 1960s. For example, Stern et al. (1964) have detected the pigment biosynthesis in qualitative and quantitative numbers during the development of chloroplast, and Epstein and Schiff (1961) have captured the process images from proplastids to develop into mature chloroplasts by electron and fluorescence microscopy in 72 h. The development of *E. gracilis* chloroplasts is a process tightly time regulated by light exposure. *Euglena* has a reduced content of chloroplast genes for photosynthetic and non-photosynthetic activities relative to the land plants. The chloroplast genome of *E. gracilis* was the first chloroplast genome completely sequenced (Hallick et al., 1993), including 55 known genes for components of the chloroplast 70S ribosomes, tRNAs, and translation factors; 27 genes for components of the thylakoid membranes, the chloroplast ATP synthase complex, or the CO_2_-fixing enzyme RUBISCO; five ORFs identified by similarity to other chloroplast ORFs; and 10 other ORFs of unknown function. *E. gracilis* nuclei encode and regulate more than 90% of proteins in chloroplasts such as light-harvesting complex proteins (Koziol and Durnford, 2008). The critical time points of chloroplast development are on the initiated time in 12 h and the stable time in 72 h for the poorly organelle development in the dark to mature functional chloroplasts after light exposure. The chlorophyll synthesis of *E. gracilis* is light-induced and exhibits a 12 h lag period for the rapid synthesis of the photosystem I (PSI) reaction centers, while roughly an 18–24 h lag period is recorded for the trigger of photosystem II (PSII) reaction centers (Stern et al., 1964; Yi et al., 1985), and the synthesis rate of chloroplast related-proteins is almost stable after 72 h of light exposure (Monroy et al., 1987).

The chloroplast development and nucleus-encoded protein synthesis induced by light are controlled at the translational level rather than transcript level (Schwartzbach, 2017), however, few reports focus on the development of chloroplast of *E. gracilis* by proteome, probably due to a lack of a high-quality assembled genome. In this study, we employed the long-term dark-adapted WT and an Ofloxacin permanently bleached mutant of *E. gracilis* B2 for a comparative proteomic study. Both WT and B2 have continuously been cultured in the dark for six months. To investigate the proteome differences of the development of *E. gracilis* chloroplast, both WT and B2 cells were compared under the light exposure at the following critical time points of 0, 12, and 72 h. We found that both *E. gracilis* WT and B2 strains exhibited light response and light regulation at the protein level. The proteins related to photosynthesis were up-regulated over time in both WT and B2. Although the random deletion of the plastid genome in B2 prevented its chlorophyll synthesis, proteomic data showed that it may have a complete function of the photosynthetic system. The main metabolic activities for the utilization of carbon and energy in WT were up-regulated, while the proteins with calcium ion binding, cell cycle, and non-photosynthetic carbon fixation were down-regulated in B2. The study provided a new understanding of *E. gracilis* chloroplast development during the re-greening process and compared the similarity and differences between WT and B2 at the proteome level.

## Materials and Methods

### Growth Conditions and Sampling

The *E. gracilis* Z strain (WT) and *E. gracilis* Oflaxocin bleached mutant strain (B2) were grown in EM medium with 1% ethanol under dark, which, for preparation,was inoculated into the refresh medium every 10 days for approximately 7 months. The WT was purchased from CCAP (Culture Collection of Algae and Protozoa, United Kingdom), and the acquisition of B2 has followed the protocol as described in Qin et al. (2020). For the light treatments, both WT and B2 cells were exposed to a light intensity of approximately 50 μmol photons·m^−2^·s^−1^ in an illuminating incubator at 25°C without shaking. The initial density of the cell was 1×10^6^ cell/ml from dark cultures in a 250 ml conical flask containing 100 ml, and total cells were collected by centrifugation at 500 *g* for 5 min at 0, 12, and 72 h after light exposure with three duplicates, respectively. The samples were stored at −80°C for protein extraction.

### Fluorescence Imaging

To detect *E. gracilis* chloroplast development, the images were viewed and captured using a Leica DMI 3000 B inverted fluorescence microscope epi-fluorescence microscope. The blue argon laser (488 nm) was chosen as the exciting light and the auto-fluorescence emitted light of chloroplasts was collected between 660 and 731 nm.

### Protein Extraction and Digestion

Protein extraction was performed as described previously (Coman et al., 2016). Briefly, the frozen samples were thawed and approximately 400 μl of pre-cooled acetone, under −40°C, was added for precipitation for 4 h. Then, the pellet of each sample was collected by centrifugation at 6,000 *g* at 4°C for 10 min, and 300 μl of 80% acetone was added to rinse the pellet with a centrifuge at 6,000 *g* for 10 min. After repeating this step twice, 100 μl of HEPES-SDC (Sigma, United States) was added to dissolve the protein and ultrasonication was performed on ice for 2 min. Finally, the supernatant was collected after centrifugation at 6,000 *g* for 10 min. For digestion, each sample was added with pre-cooled acetone at a 5-fold volume, shaken on ice for 30 min and the proteins were precipitated at −20°C for 4 h. The supernatant was removed carefully after centrifugation at 6,000 *g* at 4°C for 10 min, 200 μl of pre-cooled 80% acetone was added and the pellet was collected after centrifugation at 6,000 *g*. Then, 100 μl of 100 mM HEPES-SDC (Sigma, United States) was added to dissolve protein and ultrasonication was performed on ice for 30 s. The dissolved protein was digested by trypsin (2 μg, Promega, United States) for 2–4 h at 42°C.

### Tandem Mass Tag Labeling and nanoLCMS/MS Analysis

The digested samples were added with 15 μl of TMT (Thermo Scientific, United States) and incubated at room temperature for 1 h, then added with 2 μl of 5% hydroxylamine and incubated again at room temperature for 15 min. The collected fractions were SDC cleanup with 2% trifluoroacetic acid (TFA, Sigma, United States) and peptide desalting with desalting buffer work solution Acetonitrile (ACN, ANPEL Laboratory Technologies, China). The samples were performed in XBridge BEH C18 XP Column (Waters, United States).

For nanoLCMS/MS analysis, 1 μg of total peptides for each sample were conducted on a nano-UPLC (EASYnLC1200) coupled to a Q Exactive HFX Orbitrap instrument (Thermo Fisher Scientific). A reversed-phase column (100 μm ID ×15 cm, Reprosil-Pur 120 C18-AQ, 1.9 μm, Dr. Math) was used. Mobile phases A were H_2_O with 0.1% formic acid (FA, Sigma, United States), 2% ACN, and phase B were 80% ACN, 0.1% FA. The samples were separated with a gradient of 90 min at a flow rate of 300 nL/min in gradient A, while gradient B: 2–5% for 2 min, 5–22% for 68 min, 22–45% for 16 min, 45–95% for 2 min, 95% for 2 min. Finally, data-dependent acquisition (DDA) was performed in profile and positive mode with an Orbitrap analyzer.

### Proteomic Data Analysis

After data quality control, protein sequences were searched against their species-level UniProt FASTA databases (UniProt-Euglenophyceae-107469-2021-03), and the proteomics data have been deposited to the ProteomeXchange Consortium with the dataset identifier PXD030414. Time-series analysis was conducted using the maSigPro package (v1.54) (Conesa and Nueda, 2018). Differentially expressed proteins (DEPs) among groups were filtered with 1) more than one unique peptide, 2) foldchange more than 1.5 or less than 0.67, and 3) *p*-value less than 0.05. COG (Cluster of Orthologous Groups of proteins) annotations were obtained by eggNOG (v5.0.0) and eggNOG-mapper (v2) using the protein sequences (Huerta-Cepas et al., 2019; Cantalapiedra et al., 2021). GO (gene ontology) annotations were extracted from the UniProt database (20210419). KEGG (Kyoto Encyclopedia of Genes and Genomes) annotations were obtained by BlastKOALA and GhostKOALA (Kanehisa et al., 2016), while the annotation was used only if the result was the same in at least two databases. GO and KEGG enrichment was conducted using clusterProfiler (v3.10.1) with the *enricher* function. Venn plots were generated using Venny (v2.1) (Yu et al., 2012; Oliveros, 2016). All the other figures were generated in R (v3.5.3) using pheatmap (v1.0.12) and ggplot2 (v3.2.1) packages.

## Results

### The Development of *E. gracilis* Chloroplasts

**FIGURE 1:**
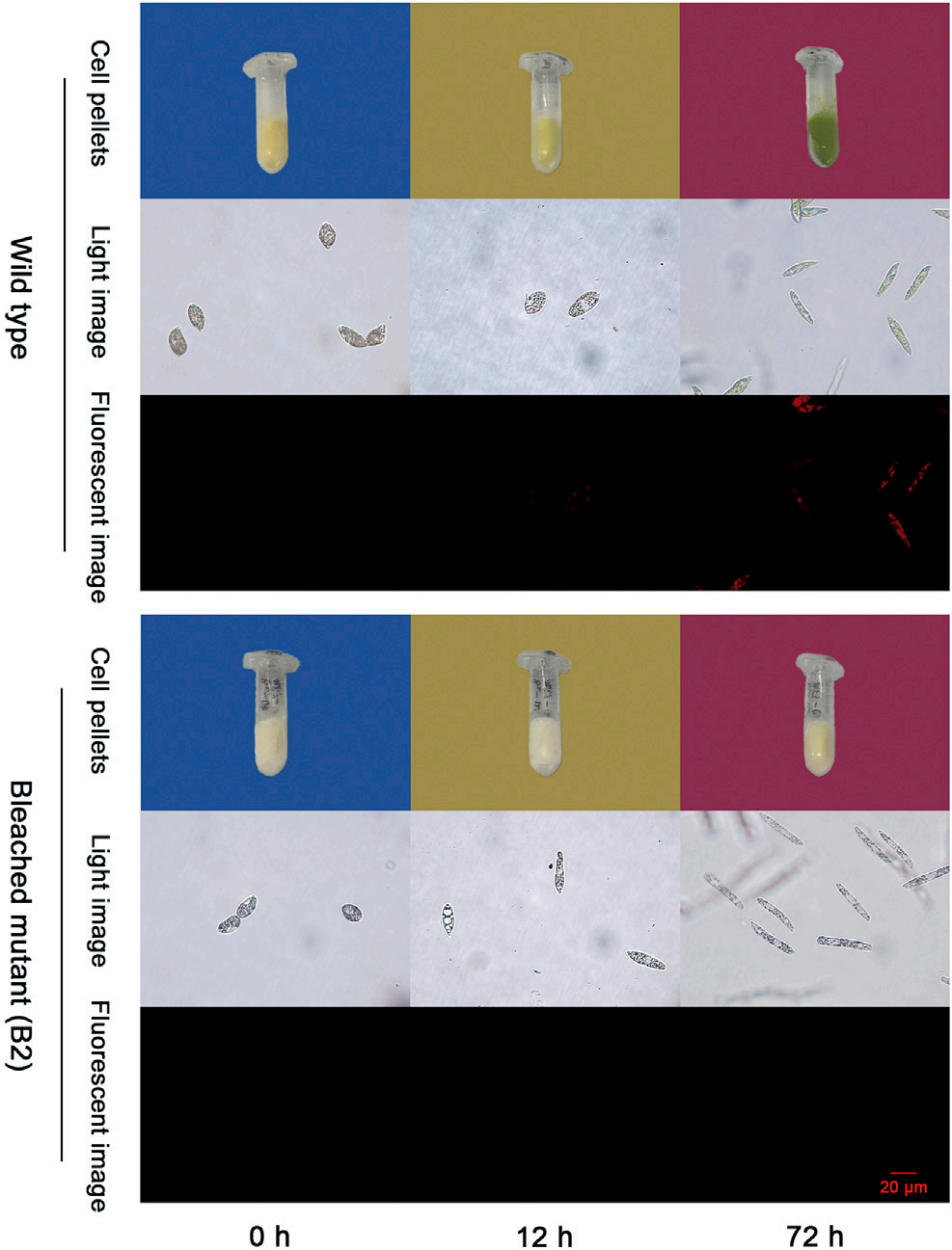
Photos of chloroplast development in wild type (WT) and bleached mutant (B2) of *E. gracilis* after light exposure restoration. Two groups (WT and B2) that included cell pellets images and microscopy images from visible light and fluorescent light were shown at the time point of 0, 12, and 72 h, respectively.

As shown in Figure 1, the color of WT that grew in the conical flask turns white to green when exposed to light from 0 to 72 h and no color changes were observed in B2 culture bottles after 72 h of light exposure. The phenomena is also consistent with the light images under microscopy. Moreover, the red fluorescence intensity of chloroplasts of WT was significantly associated with exposure time, while no fluorescence is visible for the bleached mutant cells. The red fluorescence intensity was enhanced over time in WT compared to B2. Results indicated that the chloroplast functional integrity is affiliated with light exposure, showing a time-dependent pattern, and B2 lost the ability to accumulate chlorophyll.

### The Overview of Proteomics Data

**FIGURE 2:**
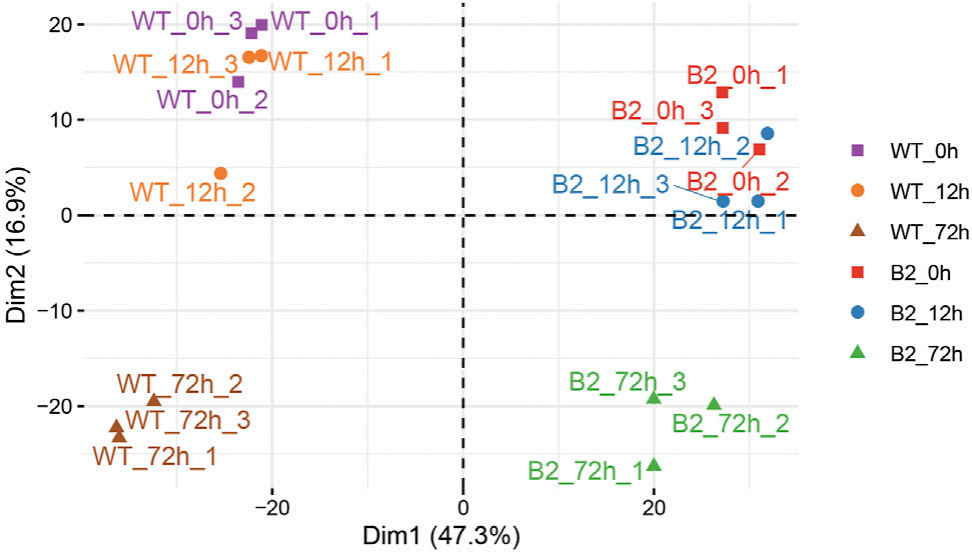
Principle component analysis (PCA) plot of proteomic samples in WT and B2. In the score plot, samples were distinguished by different shapes and colors of symbol.

In this dataset, a total of 1,572 matched peptides were obtained after removing decoy and potential polypeptides. The principle component analysis (PCA) plot showed the similarity among the samples. The difference between WT at 0 h and WT at 12 h was smaller, as well as in B2. However, at 72 h, their higher divergence was shown in the intra-group and the inter-group (Figure 2). The distribution of protein score values was shown in Supplementary Figure S1.

### The Differential Proteins Indicated by Time Serial in WT and B2

**FIGURE 3:**
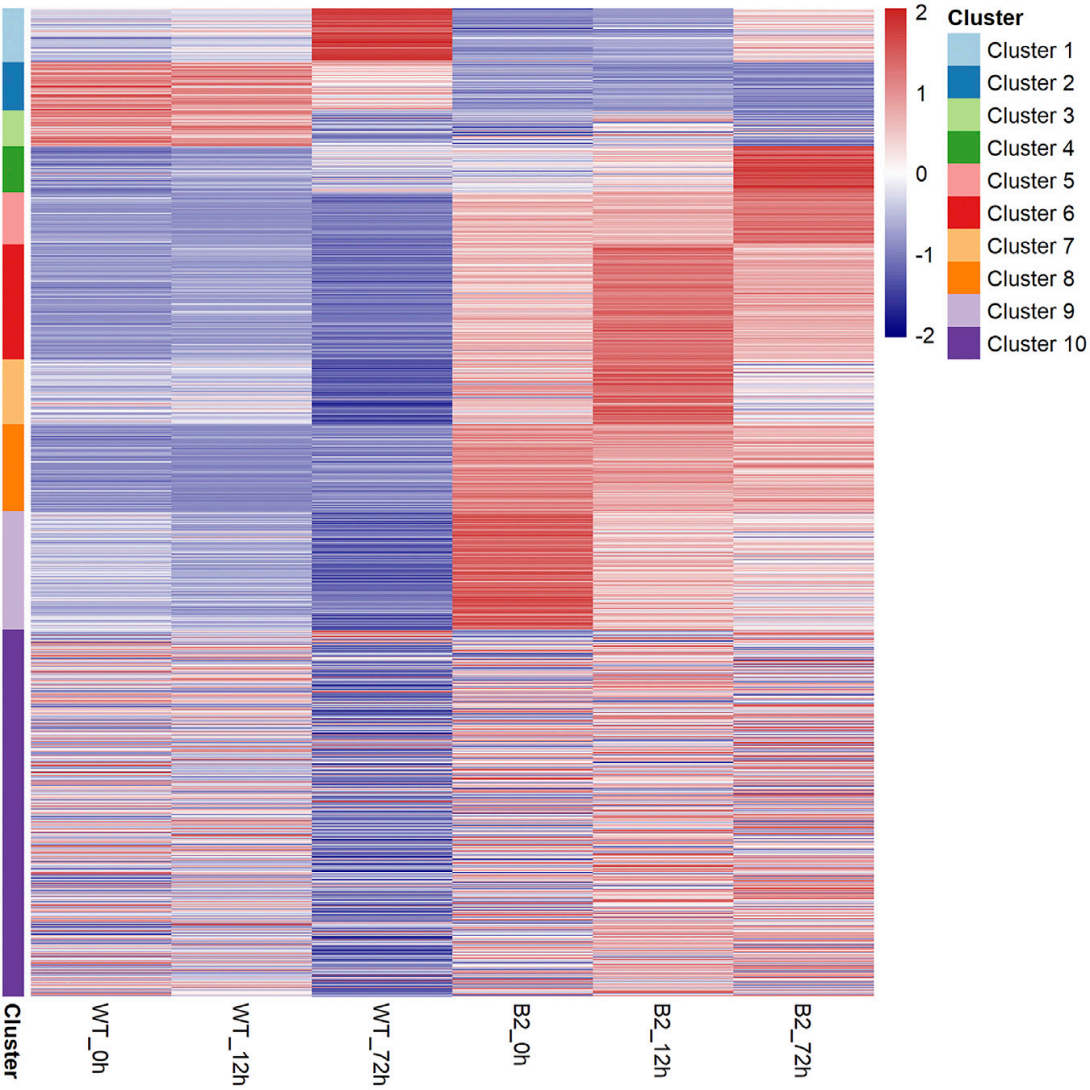
Heatmap of protein profile in WT and B2. All the proteins can be clustered as 10 groups based on their distribution similarity.

**FIGURE 4:**
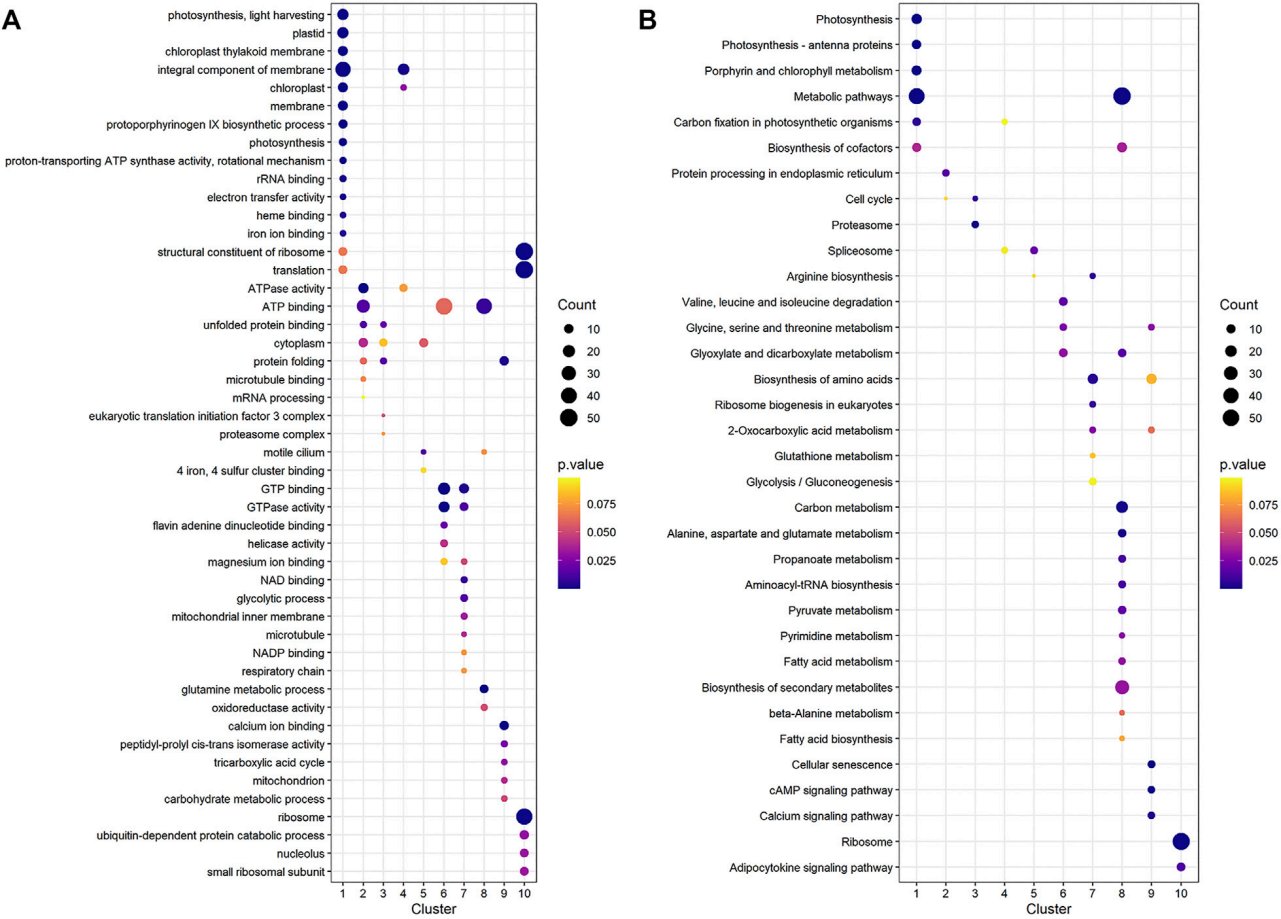
GO annotation and KEGG pathways profile of the differential protein clusters in WT and B2. **(A)** The GO annotation of the differential proteins in cluster. **(B)** KEGG pathways profile of the differential proteins in cluster. The axis of abscissa was the groups’ name and the ordinate represented the GO annotation or KEGG pathways. The size of bubbles was calculated by the number of matched proteins. *p*-value was shown on the left color bar.

To find significant protein translation profile differences between experimental groups in time course, trend analysis was conducted to the proteomics data using the maSigPro package. 988 differentially expressed peptides were screened out and classified into 9 clusters (Supplementary Figure S2), while all the non-significant peptides were classified to cluster 10. Proteins changed in WT and B2 after light re-greening was analyzed and clustered by their expression patterns in time series as shown in Figure 3. In WT, the up-regulated protein groups were largely enriched on clusters 1-3, while clusters 3-9 were down-regulated. Cluster 1 in WT-72 h was significantly up-regulated. The GO annotation of cluster1 showed that the enriched proteins were involved in photosynthesis of light-harvesting, plastid, chloroplast thylakoid membrane (an integral component of membrane), iron ion binding, chloroplast, and proton-transporting ATP synthase activity, etc. (Figure 4A). The KEGG modules or pathways of cluster1 were mostly relative to photosynthesis, such as antenna proteins, metabolic pathways, carbon fixation in photosynthetic organisms, and biosynthesis of cofactors (Figure 4B). However, cluster 10 was down-regulated in the WT at 72 h in general, and they were structural constituents of the ribosome, translation, and its pathway relevant to the ribosome (Figures 4A,B). In B2, the up-regulated proteins in 0, 12, and 72 h were in clusters 8-9, 6-7, and 4-5 respectively, while down-regulation was found in clusters 1, 2, and 3. By comparing WT with B2, clusters 1-3 were up-regulated and clusters 4-9 were down-regulated in WT and opposite results were found in B2. The GO annotation of clusters 2-5 included ATPase activity, ATP binding, cytoplasm, mRNA processing, etc., and their KEGG was mostly enriched in pathways of the cell cycle in these clusters. The GO annotation of clusters 6-9 was relative to the proteins of GTP binding, glycolytic process, mitochondrial inner membrane, NADP binding, tricarboxylic acid (TCA) cycle, and carbohydrate metabolic process, etc. (Figure 4A). Their KEGG pathways were enriched in those for energy flow, for example, biosynthesis of secondary metabolites, fatty acid metabolism, and glycolysis, etc (Figure 4B). It was apparent from these results that the *E. gracilis* chloroplast development was associated with the time point of light exposure and it was different with the bleached mutant, which lacked several key genes for chloroplast functions.

### The Differentially Expressed Proteins Among Groups

Differentially expressed proteins among samples were identified by *t*-test and filtered with 1) more than one unique peptide, 2) foldchange more than 1.5 or less than 0.67, and 3) *p*-value less than .05. The number of total-significant DEPs in WT and B2 was progressively higher with the time of light exposure, and it can be found that DEPs between 0 and 12 h for both WT and B2 were fewer than other groups, which agreed with the PCA result (Table 1). Those differential proteins were annotated to 26 orthologous COG groups (Supplementary Table S1). Thus, the groups that exhibited much more DEPs were further analyzed, including the GO annotation and KEGG pathways.

**TABLE 1:** Time course of differential expressed proteins in WT and B2.

	Total-significant	Up-regulated	Down-regulated	Not-significant
WT_72 h_vs_WT_0 h	211	148	63	1,361
WT_72 h_vs_WT_12 h	145	111	34	1,427
WT_12 h_vs_WT_0 h	9	8	1	1,563
B2_72 h_vs_B2_0 h	212	123	89	1,360
B2_72 h_vs_B2_12 h	131	94	37	1,441
B2_12 h_vs_B2_0 h	85	37	48	1,487

### The Up- and Down-Regulated GO Terms Responding to Light Treatments

**FIGURE 5:**
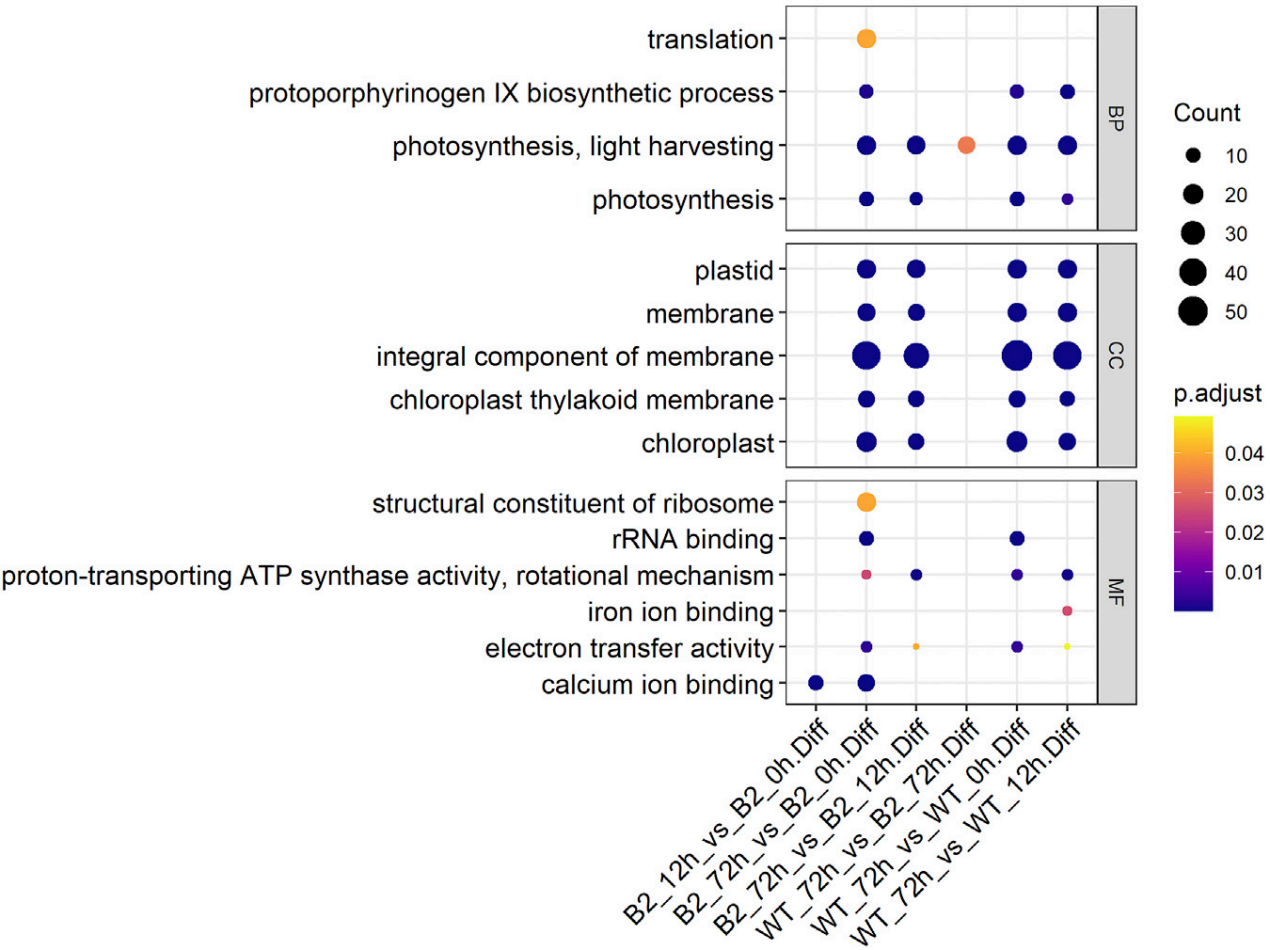
GO items in biological process (BP), cellular component (CC), and molecular function (MF) categories for the differential genes. The axis of abscissa was the groups’ name and the ordinate represented the GO annotation. The size of bubbles was calculated by the number of matched proteins. *p*-value was shown on the left color bar.

**FIGURE 6:**
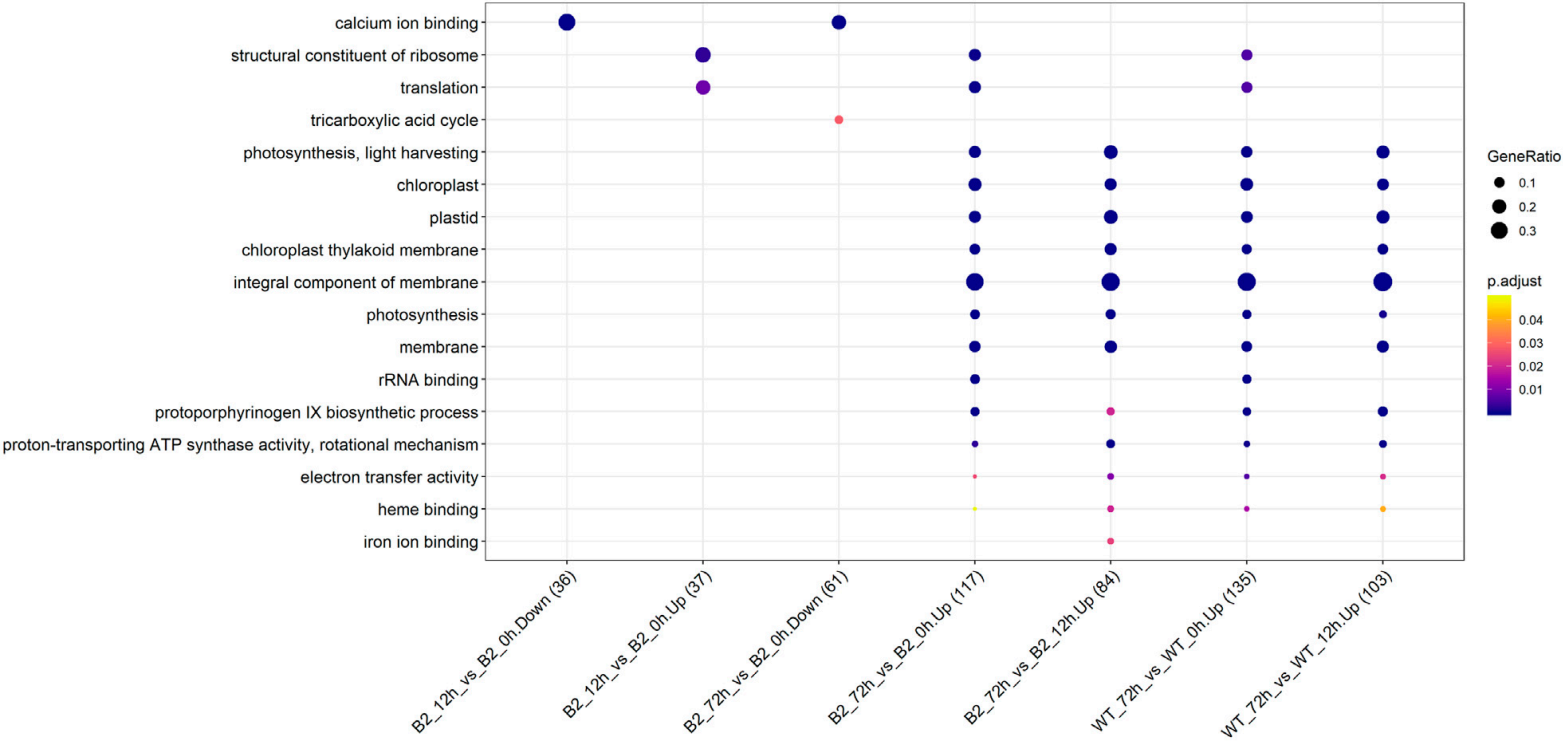
Up/down-regulated GO categories for WT and B2 during the chloroplast development. The number of matched proteins was in parentheses. The size of bubbles represented the GeneRatio of mapping GO. *p*-value was shown on the left color bar.

The GO annotation of DEPs can be divided into three main parts in the proteome, in terms of biological process (BP), cellular component (CC), and molecular function (MF) (Figure 5). There was no DEP between 0 and 12 h for the WT, while lots of DEPs were enriched on the ontology of the BP, CC, and MF relative to photosynthesis, chloroplast, and membrane after light exposure for 72 h. Interestingly, the MF proteins of calcium ion binding were most significantly differentially expressed among 0, 12, and 72 h in B2, and the catalog of calcium ion binding also was the differential part compared to WT after light exposure for 12 h. Surprisingly, the white color phenotype B2 cells with no chloroplast development harbored a large number of proteins related to chloroplast and photosynthesis revealed in this study. When compared WT with B2 by inter-group at 72 h, only the photosynthesis of light-harvesting was a differential GO term, suggesting that 12 h was a critical time point for preparing a large amount of chlorophyll accumulation (Figure 5). Besides, the GO items of calcium ion binding were down-regulated within 0 vs. 12 h of B2, while the structural constituent of ribosome and translation were up-regulated (Table 2 and Figure 6). The GO annotation about the genes of photosynthesis, chloroplast, an integral component of membrane, proton-transporting ATP synthase activity was up-regulated in the sample of WT and B2 at the time point of 72 vs 0 h and 72 vs 12 h with higher GeneRatio (the percentage of total DEPs mapped with relevant genes in the given GO term) scores respectively, and those genes were also up-regulated in the compared samples between WT and B2 at the time point of 0, 12, 72 h, respectively (Figure 6).

**TABLE 2:** The down-regulated proteins of calcium ion binding in B2.

Accession	B2_12 h vs. B2_0 h	B2_72 h vs. B2_0 h	Uniprot annotation	GhostKOALA/BlastKOALA annotation
A0A6T1PZ77	√	√	Uncharacterized protein	Hippocalcin-like protein 1
A0A6T1RQD0	√	√	Uncharacterized protein	Calmodulin
A0A6T2AAQ2		√	Uncharacterized protein	Serine/threonine-protein phosphatase 2B regulatory subunit
A0A6T2AUS4	√	√	Uncharacterized protein	Centrin-1
A0A6T2BBW3	√	√	Calmodulin	Calmodulin
A0A6T2KW10	√		Uncharacterized protein	Calmodulin
A0A6U7UI76	√	√	Uncharacterized protein	Neuronal calcium sensor
A0A6U8DBG1	√	√	Uncharacterized protein	Calmodulin
A0A6U8DJZ2	√	√	Uncharacterized protein	Centrin-1
A0A6U8KVF6		√	Uncharacterized protein	Calmodulin
B5THA2	√	√	Calmodulin 2	Calmodulin
C0LMQ2	√	√	Calmodulin 5	Calmodulin
S5R7Q0		√	Calcineurin B-like protein	Serine/threonine-protein phosphatase 2B regulatory subunit
S5RUB3	√	√	Centrin	Centrin-3

The symbol of “√” represented the down-regulation in the items over time from 0 to 72 h. The annotation results were given from the database of Uniport, BlastKOALA, and GhostKOALA.

The up-regulated and down-regulated proteins revealed by KEGG responding to light treatments.

**FIGURE 7:**
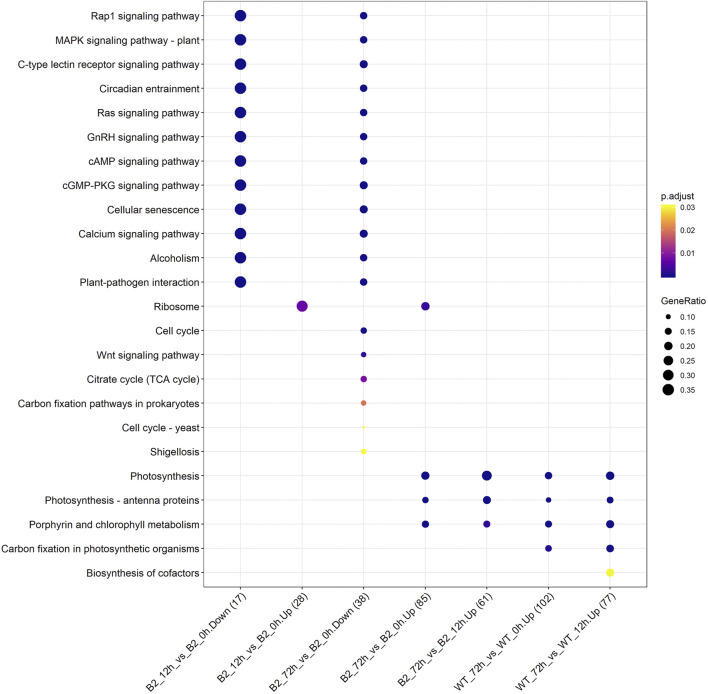
Up/down-regulated KEGG pathways for WT and B2 during chloroplast development. The number of matched proteins was in parentheses. The size of bubbles represented the GeneRatio of mapping KEGG pathways. *p*-value was shown on the left color bar.

**FIGURE 8:**
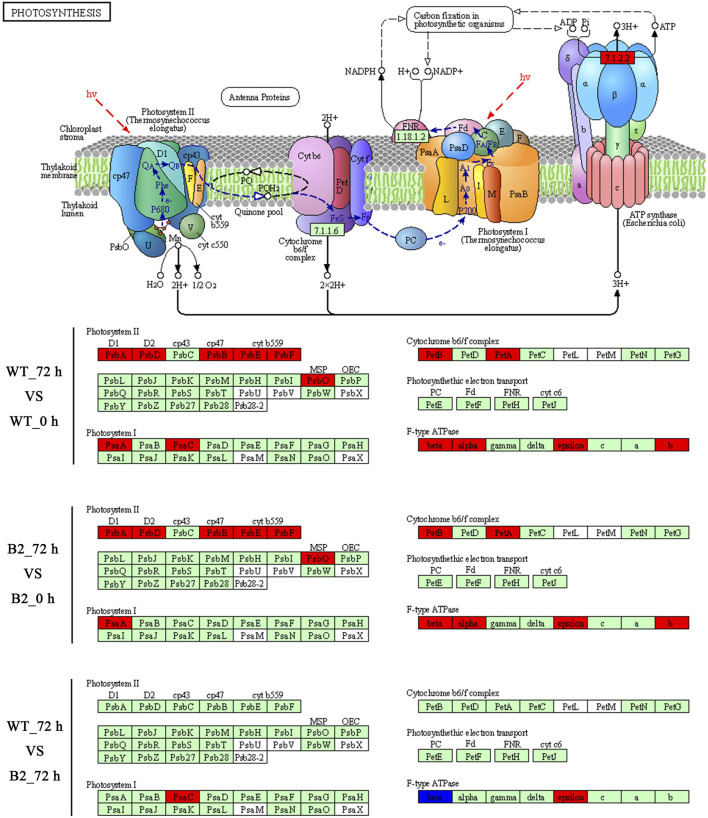
Comparison of WT and B2 in the photosynthesis KEGG pathway map after light exposure restoration. The map of the KEGG photosynthesis pathway is capable of the website on http://www.kegg.jp/pathway/map00195. The green boxes indicate the genes of encoded proteins. The red boxes indicate the genes of encoded proteins that are up-regulated, while the blue boxes are down-regulated.

The KEGG enrichment was performed to elucidate the affected pathways from poorly-developed chloroplasts to well-developed chloroplasts in WT cells and un-explored pathways in B2. Large amounts of proteins would be expected in the contribution to the physiological development of *E. gracilis* chloroplast. In WT, the proteins for carbon fixation in photosynthetic organisms were up-regulated, indicating that photosynthesis was increased with the development of the chloroplast (Figure 7 and Table 3). In B2 cells, the proteins involved in lots of signaling pathways, such as calcium signaling pathway and cAMP signaling pathway, etc. that related to the tricarboxylic acid cycle (TCA cycle), cell cycle, and non-photosynthetic carbon fixation, were down-regulated at the time point of 72 vs. 0 h (Figure 7; Tables 2, 4). For photosynthesis, the key components in the pathway are the photosystem I, photosystem II, cytochrome b6/f complex, photosynthetic electron transport, and F-type ATPase (Figure 8). At the time point of WT 72 h vs. 0 h, the proteins associated with the genes of *PsaA* and *PsaC* involved in photosystem I were up-regulated, and there were many up-regulated proteins in the photosystem II, mapping with genes of *psbA*, *psbD*, *psbB*, *psbE*, *psbF,* and *psbO*. The proteins mapped with genes of cytochrome b6/f complex (*petB* and *petA*) and a couple of proteins expressed by the genes of F-type ATPase (*β*, *α*, and *ɛ*) also were up-regulated. Interestingly, the proteins of photosynthesis in B2 have virtually identical regulation patterns with the WT during light exposure. By comparing the WT and B2 group after 72 h of light exposure, most of the differentially expressed proteins were not enriched in the photosynthesis pathway, however, the related proteins associated with the genes of *PsaC* in photosystem I and *ɛ* F-type ATPase were up-regulated, while *β* F-type ATPase was down-regulated.

**TABLE 3:** The up-regulated proteins of carbon fixation in WT.

Accession	WT_72 h_vs_WT_0 h	WT_72 h_vs_WT_12 h	Uniprot annotation	GhostKOALA/BlastKOALA annotation
A0A0G3VP19	√	√	Ribulose bisphosphate carboxylase large chain	Ribulose-bisphosphate carboxylase large chain
A0A0S3IUP6	√	√	Glyceraldehyde-3-phosphate dehydrogenase (Fragment)	Glyceraldehyde-3-phosphate dehydrogenase (NADP+) (phosphorylating)
A0A0S3IUU1	√		Triosephosphate isomerase (Fragment)	Triosephosphate isomerase
A0A1B0UKY2		√	Ribulose bisphosphate carboxylase large chain	Ribulose-bisphosphate carboxylase large chain
A0A223FM28	√	√	Ribulose bisphosphate carboxylase large chain	Ribulose-bisphosphate carboxylase large chain
A0A6T2A6Q9	√	√	Phosphoglycerate kinase (Fragment)	Phosphoglycerate kinase
A0A6T2AV11	√		Fructose-bisphosphatase	Fructose-1,6-bisphosphatase I
A0A6U8NXJ7		√	Phosphoglycerate kinase (Fragment)	Phosphoglycerate kinase
A0A6T2G8Q6	√	√	Fructose-bisphosphate aldolase (Fragment)	Fructose-bisphosphate aldolase, class I
A3QSS1	√	√	Fructose-bisphosphatase	Sedoheptulose-bisphosphatase
A3QSS7	√	√	Fructose-bisphosphatase	Fructose-1,6-bisphosphatase I
P16881	√	√	Ribulose bisphosphate carboxylase small chains, chloroplastic	Ribulose-bisphosphate carboxylase small chain
Q0IKM1	√	√	Transketolase	Transketolase
Q24LT0	√	√	Phosphopentokinase	Phosphoribulokinase
Q42728	√		Fructose-bisphosphate aldolase	Fructose-bisphosphate aldolase, class I
Q66PT3	√	√	Phosphoglycerate kinase (Fragment)	Phosphoglycerate kinase

The symbol of “√” represented the up-regulation in the items at the time point of 72 h. The annotation results were given from the database of Uniport, BlastKOALA, and GhostKOALA.

**TABLE 4:** The down-regulated proteins in B2 at the time point of 72 vs. 0 h.

Accession	KEGG group	Uniprot annotation	GhostKOALA/BlastKOALA annotation
A0A6T2CBD9	TCA cycle	Aconitate hydratase	Aconitate hydratase
A0A6T2G4I3		Succinate--CoA ligase [ADP-forming] subunit beta, mitochondrial	Succinyl-coa synthetase beta subunit
A0A6U7T5I6		Uncharacterized protein (Fragment)	Isocitrate dehydrogenase
A0A6U7TN79		2Fe-2S ferredoxin-type domain-containing protein (Fragment)	Succinate dehydrogenase (ubiquinone) iron-sulfur subunit
A0A6U7U1Z3		(2R,3S)-2-methylisocitrate dehydratase (Fragment)	Aconitate hydratase 2/2-methylisocitrate dehydratase
A0A6T1W9H8	Cell cycle	CULLIN_2 domain-containing protein	Cullin 1
A0A6T1XVY5		14_3_3 domain-containing protein	14-3-3 protein epsilon
A0A6T2BUY8		Uncharacterized protein	S-phase kinase-associated protein 1
A0A6T2GVH2		14_3_3 domain-containing protein	14-3-3 protein epsilon
A0A6U7TT72		RING-type domain-containing protein	E3 ubiquitin-protein ligase RBX1
A0A6T2CBD9	non-photosynthetic carbon fixation	Aconitate hydratase	Aconitate hydratase
A0A6T2J8J2		Pyruvate, phosphate dikinase	Pyruvate, orthophosphate dikinase
A0A6U7T5I6		Uncharacterized protein (Fragment)	Isocitrate dehydrogenase
A0A6U7U1Z3		(2R,3S)-2-methylisocitrate dehydratase (Fragment)	Aconitate hydratase 2/2-methylisocitrate dehydratase

The up-regulation items of TCA, cycle, cell cycle, and non-photosynthetic carbon fixation were shown at the time point of 72 vs. 0 h. The annotation results were given from the database of Uniport, BlastKOALA, and GhostKOALA.

## Discussion

*E. gracilis* is one of the most widely studied model species for chloroplast development, due to the easy loss and secondary endosymbiosis of chloroplasts by the ancestor of *Euglena* and green algae (Zakryś et al., 2017). The process of the acquisition of foreign chloroplast for *E. gracilis* integrates the new organelle into a cell, as well as the new genes into nuclear genomes, which were gifted with new coding proteins and metabolic pathways. In recent decades, much research has been conducted related to the *E. gracilis* chloroplast. For example, in 1993 the chloroplast genome of *E. gracilis* was entirely sequenced and the size of the plastid genome was 143 kbp and encoded 97 proteins and genes (Hallick et al., 1993). Novák Vanclová et al. (2020) revealed metabolic quirks and a colorful history of the secondary plastid of *E. gracilis* by proteome. However, the chloroplast is dispensable for *E. gracilis* and is easily abandoned under various abiotic conditions such as antibiotics, heat, ultraviolet light, and so on (Gibor and Granick, 1962). The green *E. gracilis* cells, that grow in the dark for an estimated two weeks, can be turned temporarily colorless (Qin et al., 2020). It seems that the existence of chloroplasts in *E. gracilis* cells are wasteful in the absence of light and light is the crucial regulating factor for their chloroplast development. The development of chloroplast in *E. gracilis* was regulated at the translational level rather than the transcriptional level (Schwartzbach, 2017). Thus, the present study aimed to explore the relationship between *E. gracilis* chloroplast development and light treatment from the dark by proteome, using both dark-adapted WT and B2 cells.

WT *E. gracilis* cells respond to light and exhibit light regulation with a complex and complicated process (Schwartzbach and Shigeoka, 2017). In general, dark-adapted WT chloroplasts exist as an estimated ten small proplastids, lacking internal membranes and containing some prolamellar bodies, and can be recovered after light exposure (Ahn et al., 2018). The time point of 72 h is enough for proplastids to turn into mature functional chloroplasts, changing from white cells to green cells (Figure 1). This can also be proven by the two-dimensional gel electrophoresis for the detection of chloroplast pigment proteins by time serial in *E. gracilis* (Monroy et al., 1987). It is obvious that the proteins related to photosynthesis and light-harvesting protein groups finally can be up-regulated in WT after light exposure (Figure 4). Though many investigations were carried out in WT to study the chloroplast development under light exposure, there is no report about the bleached mutants in this case. The most well-known example of colorless euglenid is *E. longa*, a species very close relative to *E. gracilis*. It lost most of the photosynthetic genes with dormant plastids (Gockel and Hachtel, 2000). B2 still has residual chloroplasts and absences many genes in plastid genomes but not all of them (Qin et al., 2020). Those residual plastids can be observed under transmission electron microscopy (Kivic and Vesk, 1974). As the first study, we found that B2 also had a similar response to light, but the level of the differential expression was relatively lower than WT (Figure 5). This can explain why B2 still had the light-regulated properties in this study.

The light-induced chloroplast development was time-dependent. In WT, there existed a 12 h lag period for the activation of the photosynthesis system resulting in no significant difference between the WT 0 h and WT 12 h. In previous studies, the chlorophyll accumulation exhibited a 12 h lag period by photo induction of LHCP (light harvesting chlorophyll a/b binding protein of photosystem) II accumulation (Schwartzbach et al., 1976; Bouet et al., 1986; Weiss et al., 1988). We found that the number of DEPs was increased and the changed proteins are mainly related to cell metabolisms, such as ATP binding, GTPase activity, tricarboxylic acid cycle, pyruvate metabolism, carbon metabolism, etc. after a time point of 12 h (Figures 3, 4). Perhaps, since the enzyme activity of the photosynthetic system was disrupted by the functional insufficiency of the residual chloroplast, the B2 cells can act with light exposure but are not prepared for chlorophyll accumulation. In addition, the down-regulation of some proteins (ATP binding, unfolded protein binding, and protein folding) or other unknown functional proteins involved in chlorophyll accumulation may lead to different responses for light exposure over time in B2 (cluster 2 and 3 in Figure 4A). Thus, a significantly lower or different light-responsive proteome could be expected in B2 compared to WT.

However, the loss of many chloroplast genes in B2 (Qin et al., 2020) seemed to not affect the integrity of the encoded proteins in the photosynthesis systems. The results of the KEGG photosynthesis pathway for photosynthetic electron transport (including PSI, PSII, cytochrome *b6f* (Cyt*b6f*), and ATP synthase), showed the proteins relevant to their expressed genes and pathways (Figures 6, 7; Table 3). The CO_2_ was fixated in PS I by providing the negative redox, while molecular oxygen and H^+^ were produced in PSII by oxidizing water. The Cyt*b6f* complex afforded a pathway for plastoquinone and plastocyanin, which allowed the electrons to be a shuttle between the PSI and PSII. At the same time, ATP synthase was driven by the proton gradient (Bohne et al., 2016). All components of the functional genome of chloroplast guarantee the photosynthetic system’s proper function. By comparing WT to B2 at 72 h, it was common that the proteins associated with the gene of *PsaC* in PSI was up-regulated (Figure 8). Although the gene of *PsaC* responded positively with light re-greening both in WT and B2, the synthesis rate of relevant protein was high to WT rather than B2. The cells of WT underwent much more cell division rapidly than B2 in the same growth condition, due to the incomplete chloroplast genome of B2 (Qin et al., 2020). The F-type ATPase was assembled by five globular proteins, including *α*, *β*, *γ*, *δ* and *ɛ* as a whole (Yagi et al., 2007). It was difficult to clearly explain the relationship between one part of the globular protein and its physiological role. In Qin et al. (2020) study, 50 chloroplast-relevant genes were examined for B2 (OflB2 in the reference article) by PCR, and 37 involved genes were lost, but all of them were detected in WT. This indicated that B2 only retained parts of the chloroplast genome. Indeed, the results of chloroplast genes’ annotation by proteome showed that all the proteins related to photosynthesis identified in WT were also detected in B2, but those proteins in WT were significantly up-regulated compared to B2 (Figure 6). It was implied that the functional genes of chloroplast in B2 were still maintained and properly functioned at the protein level. However, the relevant genome of chloroplast for B2 was lost randomly (Qin et al., 2020). That was potentially attributed to the different detection methods between the genome level and proteome level. Further studies will be required to elucidate this phenomenon.

The major metabolic activities related to chloroplast development in *E. gracilis* were those required for the use of carbon and energy to ensure proliferation. In previous studies, *E. gracilis* has been perceived as a more diverse metabolic pathway for using carbon. For example, the C2 compounds of the glyoxylate pathway have three fates, including the regulation of the TCA cycle and the formation of serine *via* glycine and formate (Hasan et al., 2019), and the C5 pathway of aminolevulinate synthesis and amino acid metabolism also have been reported in recent years (Vanclová et al., 2019), etc. *Euglena* can utilize those organic compounds to synthesize and store carbohydrate paramylum in the dark, whereas most of them would be degraded induced by light. The degradation of paramylum will produce inhibitors and uncouples that block the chloroplast development of *E. gracilis* (Fong and Schiff, 1977; Klein et al., 1972). As we know, the chloroplast genomes encode many important proteins of photosynthesis needed for CO_2_ fixation. All of these proteins or pathways were light-regulated and involved in the metabolic activities to the positive feedback for the photosynthesis abilities of *E. gracilis*. Thus, with the increased degradation effect of respiration inhibitors, the capacity of CO_2_ fixation was enhanced by more chlorophyll synthesis in the light. Besides, the proteins or pathways involved in calcium ion binding, cell cycle, and non-photosynthetic carbon fixation were down-regulated in B2 (Tables 2, 4). That might be the reason for the incomplete development of chloroplast in B2. By comparing WT and B2, the speed of cell division, the biomass, and the cell motility in WT were more effective than that of B2 during the light exposure (Qin et al., 2020). Calcium-binding protein regulates plenty of different targeting proteins and plays an important role in the plants’ signaling pathway (Pirayesh et al., 2021). These proteins can regulate the flagellar moving pattern and motility through the calcium ion signaling pathway (Watanabe and Iseki, 2005). The down-regulation of calcium ion proteins may result in insensitive photo-movement responses for B2, which showed the difference to WT as observed in the study (Figure 5). Chloroplast division occurs simultaneously with nuclear division and requires high levels of pigment and protein synthesis to maintain the photosynthetic capacity of newly divided chloroplasts. The down-regulation of cell cycle proteins or pathways could be the photo-induced cell cycle arrest. Cell cycle progressions are required for photo-induced commitments (Hagiwara et al., 2001). The non-photosynthetic carbon fixation is putative in the study. Many proteins assigned to aconitate hydratase, pyruvate, phosphate dikinase, and isocitrate dehydrogenase were down-regulated. Those proteins are involved in carbon metabolism. The most executive function of chloroplast is carbon fixation and offers triose phosphates to the biosynthesis of amino acids, carbohydrates, and fatty acids (Kamikawa et al., 2017; Hasan et al., 2019). Further studies will be required to verify the complicated mechanism of non-photosynthetic carbon fixation in *Euglena*.

## Data Availability

The datasets presented in this study can be found in online repositories. The mass spectrometry proteomics data have been deposited to the ProteomeXchange Consortium *via* the PRIDE partner repository with the dataset identifier PXD030414.
