# Supplementary material for: Proteomic Responses of Dark-Adapted Euglena gracilis and Bleached Mutant Against Light Stimuli
Source: Front Bioeng Biotechnol. 2022 Mar 3;10:843414. doi: 10.3389/fbioe.2022.843414 (PMC8927018; doi:10.3389/fbioe.2022.843414)
Supplement: Supplementary file 1 [file Table1.DOCX]

Supplementary Table 1. The COG functional classification and its abundance of the up/down-regulated proteins.

| Compare | Regulation | A | B | C | D | E | F | G | H | I | J | K | L | M | N | O | P | Q | R | S | T | U | V | W | X | Y | Z |
| --- | --- | --- | --- | --- | --- | --- | --- | --- | --- | --- | --- | --- | --- | --- | --- | --- | --- | --- | --- | --- | --- | --- | --- | --- | --- | --- | --- |
| WT_12h_vs_WT_0h | All | 0 | 0 | 0 | 0 | 0 | 1 | 1 | 1 | 2 | 0 | 0 | 0 | 0 | 0 | 0 | 0 | 2 | 0 | 2 | 0 | 1 | 0 | 0 | 0 | 0 | 0 |
|  | Up | 0 | 0 | 0 | 0 | 0 | 1 | 1 | 1 | 2 | 0 | 0 | 0 | 0 | 0 | 0 | 0 | 2 | 0 | 2 | 0 | 1 | 0 | 0 | 0 | 0 | 0 |
|  | Down | 0 | 0 | 0 | 0 | 0 | 0 | 0 | 0 | 0 | 0 | 0 | 0 | 0 | 0 | 0 | 0 | 0 | 0 | 0 | 0 | 0 | 0 | 0 | 0 | 0 | 0 |
| WT_72h_vs_WT_0h | All | 5 | 0 | 41 | 2 | 11 | 9 | 14 | 13 | 10 | 28 | 3 | 2 | 0 | 0 | 16 | 1 | 8 | 0 | 20 | 1 | 8 | 0 | 0 | 0 | 1 | 2 |
|  | Up | 1 | 0 | 34 | 0 | 11 | 7 | 13 | 12 | 5 | 23 | 1 | 1 | 0 | 0 | 10 | 1 | 8 | 0 | 13 | 0 | 6 | 0 | 0 | 0 | 1 | 1 |
|  | Down | 4 | 0 | 7 | 2 | 0 | 2 | 1 | 1 | 5 | 5 | 2 | 1 | 0 | 0 | 6 | 0 | 0 | 0 | 7 | 1 | 2 | 0 | 0 | 0 | 0 | 1 |
| WT_72h_vs_WT_12h | All | 3 | 0 | 35 | 0 | 7 | 1 | 8 | 14 | 3 | 13 | 1 | 1 | 0 | 0 | 14 | 1 | 7 | 0 | 10 | 0 | 9 | 0 | 0 | 0 | 0 | 0 |
|  | Up | 1 | 0 | 31 | 0 | 7 | 1 | 8 | 13 | 2 | 13 | 0 | 1 | 0 | 0 | 8 | 1 | 7 | 0 | 7 | 0 | 5 | 0 | 0 | 0 | 0 | 0 |
|  | Down | 2 | 0 | 4 | 0 | 0 | 0 | 0 | 1 | 1 | 0 | 1 | 0 | 0 | 0 | 6 | 0 | 0 | 0 | 3 | 0 | 4 | 0 | 0 | 0 | 0 | 0 |
| WT_0h_vs_B2_0h | All | 28 | 2 | 53 | 11 | 37 | 15 | 25 | 26 | 26 | 58 | 11 | 8 | 4 | 4 | 68 | 5 | 13 | 0 | 48 | 34 | 38 | 0 | 0 | 0 | 1 | 31 |
|  | Up | 4 | 0 | 21 | 0 | 4 | 2 | 3 | 4 | 1 | 14 | 2 | 1 | 1 | 0 | 20 | 0 | 1 | 0 | 8 | 6 | 5 | 0 | 0 | 0 | 0 | 4 |
|  | Down | 24 | 2 | 32 | 11 | 33 | 13 | 22 | 22 | 25 | 44 | 9 | 7 | 3 | 4 | 48 | 5 | 12 | 0 | 40 | 28 | 33 | 0 | 0 | 0 | 1 | 27 |
| WT_12h_vs_B2_12h | All | 37 | 4 | 50 | 6 | 40 | 19 | 30 | 25 | 29 | 58 | 17 | 12 | 5 | 4 | 70 | 4 | 13 | 0 | 54 | 32 | 35 | 1 | 0 | 0 | 1 | 22 |
|  | Up | 5 | 0 | 16 | 0 | 2 | 2 | 5 | 4 | 1 | 10 | 4 | 1 | 1 | 0 | 20 | 0 | 2 | 0 | 9 | 5 | 5 | 0 | 0 | 0 | 0 | 3 |
|  | Down | 32 | 4 | 34 | 6 | 38 | 17 | 25 | 21 | 28 | 48 | 13 | 11 | 4 | 4 | 50 | 4 | 11 | 0 | 45 | 27 | 30 | 1 | 0 | 0 | 1 | 19 |
| WT_72h_vs_B2_72h | All | 46 | 6 | 64 | 7 | 30 | 16 | 24 | 21 | 26 | 67 | 15 | 8 | 6 | 5 | 81 | 3 | 11 | 0 | 63 | 42 | 38 | 0 | 0 | 0 | 1 | 27 |
|  | Up | 5 | 0 | 31 | 0 | 4 | 2 | 6 | 4 | 1 | 15 | 3 | 0 | 1 | 0 | 19 | 0 | 3 | 0 | 6 | 5 | 4 | 0 | 0 | 0 | 0 | 2 |
|  | Down | 41 | 6 | 33 | 7 | 26 | 14 | 18 | 17 | 25 | 52 | 12 | 8 | 5 | 5 | 62 | 3 | 8 | 0 | 57 | 37 | 34 | 0 | 0 | 0 | 1 | 25 |
| B2_12h_vs_B2_0h | All | 4 | 0 | 6 | 7 | 5 | 1 | 2 | 1 | 2 | 13 | 2 | 1 | 0 | 0 | 7 | 0 | 0 | 0 | 13 | 10 | 2 | 0 | 0 | 0 | 0 | 5 |
|  | Up | 2 | 0 | 4 | 1 | 2 | 1 | 1 | 1 | 1 | 13 | 2 | 1 | 0 | 0 | 2 | 0 | 0 | 0 | 2 | 2 | 2 | 0 | 0 | 0 | 0 | 0 |
|  | Down | 2 | 0 | 2 | 6 | 3 | 0 | 1 | 0 | 1 | 0 | 0 | 0 | 0 | 0 | 5 | 0 | 0 | 0 | 11 | 8 | 0 | 0 | 0 | 0 | 0 | 5 |
| B2_72h_vs_B2_0h | All | 6 | 1 | 39 | 8 | 13 | 1 | 9 | 11 | 4 | 26 | 7 | 1 | 0 | 0 | 18 | 1 | 5 | 0 | 22 | 12 | 6 | 0 | 0 | 0 | 0 | 8 |
|  | Up | 3 | 1 | 32 | 2 | 9 | 1 | 5 | 11 | 3 | 24 | 3 | 1 | 0 | 0 | 7 | 0 | 5 | 0 | 11 | 3 | 2 | 0 | 0 | 0 | 0 | 1 |
|  | Down | 3 | 0 | 7 | 6 | 4 | 0 | 4 | 0 | 1 | 2 | 4 | 0 | 0 | 0 | 11 | 1 | 0 | 0 | 11 | 9 | 4 | 0 | 0 | 0 | 0 | 7 |
| B2_72h_vs_B2_12h | All | 4 | 2 | 33 | 2 | 8 | 1 | 6 | 7 | 2 | 15 | 5 | 1 | 1 | 0 | 9 | 1 | 5 | 0 | 10 | 1 | 2 | 0 | 0 | 0 | 0 | 1 |
|  | Up | 3 | 1 | 32 | 1 | 3 | 0 | 4 | 7 | 0 | 11 | 4 | 0 | 0 | 0 | 7 | 0 | 4 | 0 | 8 | 1 | 1 | 0 | 0 | 0 | 0 | 1 |
|  | Down | 1 | 1 | 1 | 1 | 5 | 1 | 2 | 0 | 2 | 4 | 1 | 1 | 1 | 0 | 2 | 1 | 1 | 0 | 2 | 0 | 1 | 0 | 0 | 0 | 0 | 0 |

The categories from A to Z represented the COG functional classification as followed: A, RNA processing and modification; B, Chromatin structure and dynamics; C, Energy production and conversion; D, Cell cycle control, cell division, chromosome partitioning; E, Amino acid transport and metabolism; F, Nucleotide transport and metabolism; G, Carbohydrate transport and metabolism; H, Coenzyme transport and metabolism; I, Lipid transport and metabolism; J, Translation, ribosomal structure and biogenesis; K, Transcription; L, Replication, recombination and repair; M, Cell wall/membrane/envelope biogenesis; N, Cell motility; O, Posttranslational modification, protein turnover, chaperones; P, Inorganic ion transport and metabolism; Q, Secondary metabolites biosynthesis, transport and catabolism; R, General function prediction only; S, Function unknown; T, Signal transduction mechanisms; U, Intracellular trafficking, secretion, and vesicular transport; V, Defense mechanisms; W, Extracellular structures; X, Mobilome, prophages, transposons; Y, Nuclear structure; Z, Cytoskeleton.
