# Supplementary figures and images for: Proteomic Responses of Dark-Adapted Euglena gracilis and Bleached Mutant Against Light Stimuli
Source: Front Bioeng Biotechnol. 2022 Mar 3;10:843414. doi: 10.3389/fbioe.2022.843414 (PMC8927018; doi:10.3389/fbioe.2022.843414)

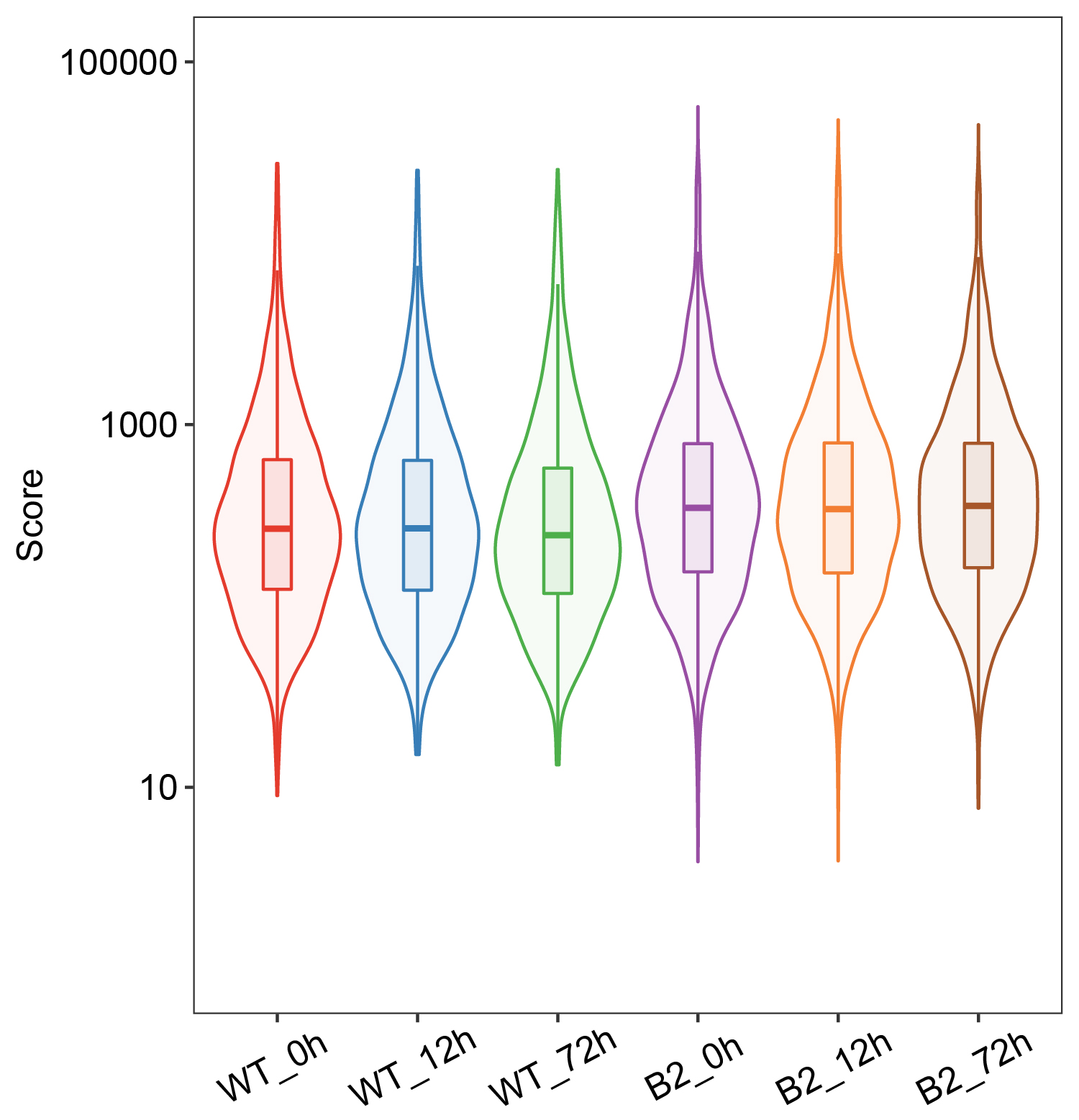

Supplement: Supplementary file 2 [file Image1.JPEG]

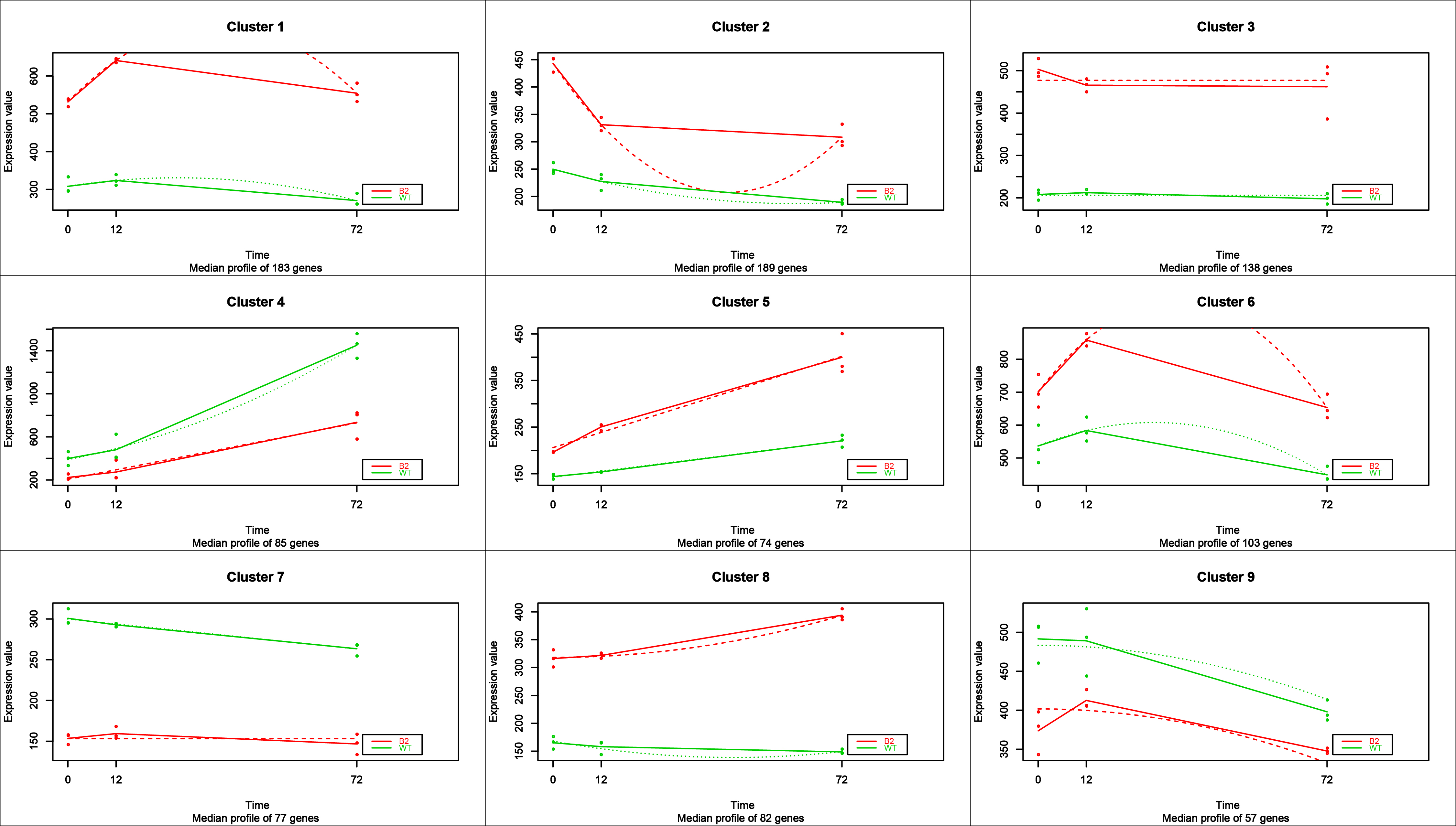

Supplement: Supplementary file 3 [file Image2.JPEG]
